# Supplementary material for: The bi-factor structure of the 17-item Hamilton Depression Rating Scale in persistent major depression; dimensional measurement of outcome
Source: PLoS One. 2020 Oct 26;15(10):e0241370. doi: 10.1371/journal.pone.0241370 (PMC7588071; doi:10.1371/journal.pone.0241370)
Supplement: S2 Data — (DOCX) [file pone.0241370.s002.docx]

S2 Data. Output of factor loadings in Table 2 models.

Item content

| DEPMD | depressed mood |
| --- | --- |
| GUILT | guilt feeling |
| SUCDB | suicidal thoughts |
| EARLY | insomnia initial |
| ISMMID | insomnia middle |
| LATE | insomnia delayed |
| WKACT | work &interests |
| RTARD | motor retardation |
| AGIT | agitation |
| ANXPSY | psychic anxiety |
| ANXSOM | somatic anxiety |
| APPT | appetite decrease |
| GENERL | tiredness |
| SEXINT | sexual interest |
| HYPCHD | hypochondriasis |
| WGTLS | weight loss |

B: baseline item

Following are items loadings for models presented in table 2

*1 Factor model

MODEL RESULTS

Two-Tailed

Estimate S.E. Est./S.E. P-Value

FB1 BY

DEPMDB 0.527 0.036 14.537 0.000

GUILTB 0.392 0.033 11.939 0.000

SUCDB 0.453 0.039 11.520 0.000

EARLYB 0.236 0.057 4.176 0.000

ISMMIDB 0.344 0.052 6.594 0.000

LATEB 0.274 0.054 5.052 0.000

WKACTB 0.492 0.043 11.475 0.000

RTARDB 0.325 0.063 5.138 0.000

AGITB 0.136 0.051 2.697 0.007

ANXPSYB 0.421 0.039 10.663 0.000

ANXSOMB 0.416 0.043 9.783 0.000

APPTB 0.365 0.047 7.846 0.000

GENERLB 0.499 0.042 11.953 0.000

SEXINTB 0.290 0.052 5.567 0.000

HYPCHDB 0.197 0.069 2.858 0.004

WGTLSB 0.215 0.059 3.669 0.000

*2 Factor model

MODEL RESULTS

Two-Tailed

Estimate S.E. Est./S.E. P-Value

FB1 BY

DEPMDB 0.577 0.038 15.038 0.000

GUILTB 0.397 0.041 9.704 0.000

SUCDB 0.462 0.041 11.290 0.000

EARLYB -0.046 0.064 -0.710 0.478

ISMMIDB 0.013 0.030 0.417 0.676

LATEB 0.010 0.034 0.287 0.774

WKACTB 0.453 0.043 10.433 0.000

RTARDB 0.403 0.060 6.733 0.000

AGITB 0.023 0.058 0.396 0.692

ANXPSYB 0.480 0.043 11.110 0.000

ANXSOMB 0.388 0.047 8.163 0.000

APPTB 0.151 0.058 2.586 0.010

GENERLB 0.409 0.051 8.068 0.000

SEXINTB 0.285 0.060 4.754 0.000

HYPCHDB 0.249 0.096 2.599 0.009

WGTLSB -0.097 0.075 -1.291 0.197

FB2 BY

DEPMDB -0.052 0.053 -0.977 0.328

GUILTB 0.016 0.050 0.326 0.745

SUCDB 0.012 0.032 0.389 0.697

EARLYB 0.518 0.067 7.714 0.000

ISMMIDB 0.629 0.063 9.910 0.000

LATEB 0.483 0.069 6.996 0.000

WKACTB 0.105 0.063 1.663 0.096

RTARDB -0.113 0.100 -1.139 0.255

AGITB 0.214 0.082 2.602 0.009

ANXPSYB -0.067 0.073 -0.928 0.354

ANXSOMB 0.086 0.076 1.136 0.256

APPTB 0.419 0.070 5.971 0.000

GENERLB 0.201 0.075 2.697 0.007

SEXINTB 0.032 0.078 0.406 0.685

HYPCHDB -0.077 0.103 -0.744 0.457

WGTLSB 0.576 0.085 6.746 0.000

*3 Factor model

MODEL RESULTS

Two-Tailed

Estimate S.E. Est./S.E. P-Value

FB1 BY

DEPMDB 0.571 0.059 9.759 0.000

GUILTB 0.437 0.044 10.000 0.000

SUCDB 0.456 0.054 8.409 0.000

EARLYB -0.043 0.066 -0.655 0.512

ISMMIDB -0.020 0.041 -0.473 0.636

LATEB -0.002 0.039 -0.062 0.951

WKACTB 0.432 0.065 6.616 0.000

RTARDB 0.259 0.118 2.198 0.028

AGITB 0.167 0.097 1.713 0.087

ANXPSYB 0.619 0.071 8.715 0.000

ANXSOMB 0.484 0.052 9.260 0.000

APPTB 0.215 0.054 3.978 0.000

GENERLB 0.469 0.052 8.966 0.000

SEXINTB 0.297 0.063 4.743 0.000

HYPCHDB 0.361 0.109 3.299 0.001

WGTLSB 0.011 0.013 0.798 0.425

FB2 BY

DEPMDB -0.035 0.048 -0.733 0.463

GUILTB -0.007 0.041 -0.177 0.860

SUCDB 0.032 0.063 0.511 0.609

EARLYB 0.526 0.065 8.117 0.000

ISMMIDB 0.683 0.067 10.276 0.000

LATEB 0.504 0.070 7.184 0.000

WKACTB 0.140 0.058 2.408 0.016

RTARDB 0.009 0.015 0.626 0.531

AGITB 0.097 0.110 0.879 0.380

ANXPSYB -0.188 0.087 -2.155 0.031

ANXSOMB 0.004 0.062 0.059 0.953

APPTB 0.382 0.078 4.877 0.000

GENERLB 0.171 0.078 2.209 0.027

SEXINTB 0.029 0.077 0.382 0.703

HYPCHDB -0.174 0.119 -1.458 0.145

WGTLSB 0.525 0.112 4.666 0.000

FB3 BY

DEPMDB 0.323 0.091 3.565 0.000

GUILTB 0.102 0.088 1.163 0.245

SUCDB 0.239 0.083 2.884 0.004

EARLYB -0.059 0.082 -0.719 0.472

ISMMIDB 0.023 0.067 0.340 0.733

LATEB 0.006 0.050 0.112 0.911

WKACTB 0.300 0.070 4.280 0.000

RTARDB 0.629 0.085 7.412 0.000

AGITB -0.421 0.090 -4.682 0.000

ANXPSYB -0.043 0.090 -0.473 0.636

ANXSOMB -0.035 0.082 -0.422 0.673

APPTB -0.128 0.079 -1.616 0.106

GENERLB 0.019 0.042 0.454 0.650

SEXINTB 0.104 0.090 1.152 0.249

HYPCHDB -0.133 0.117 -1.130 0.258

WGTLSB -0.426 0.103 -4.133 0.000

*bi-2factor

MODEL RESULTS

Two-Tailed

Estimate S.E. Est./S.E. P-Value

HAMB BY

DEPMDB -0.072 0.088 -0.816 0.415

GUILTB -0.069 0.062 -1.113 0.266

SUCDB -0.006 0.078 -0.081 0.936

EARLYB 0.478 0.074 6.502 0.000

ISMMIDB 0.636 0.066 9.590 0.000

LATEB 0.465 0.066 7.056 0.000

WKACTB 0.111 0.081 1.364 0.173

RTARDB 0.097 0.201 0.481 0.630

AGITB -0.036 0.160 -0.225 0.822

ANXPSYB -0.302 0.096 -3.146 0.002

ANXSOMB -0.098 0.089 -1.110 0.267

APPTB 0.281 0.087 3.232 0.001

GENERLB 0.070 0.069 1.020 0.308

SEXINTB -0.008 0.077 -0.103 0.918

HYPCHDB -0.259 0.104 -2.498 0.013

WGTLSB 0.386 0.158 2.448 0.014

FB1 BY

DEPMDB 0.440 0.054 8.092 0.000

GUILTB 0.391 0.042 9.351 0.000

SUCDB 0.381 0.051 7.505 0.000

EARLYB 0.181 0.089 2.039 0.041

ISMMIDB 0.239 0.092 2.602 0.009

LATEB 0.192 0.071 2.705 0.007

WKACTB 0.380 0.059 6.489 0.000

RTARDB 0.054 0.102 0.523 0.601

AGITB 0.336 0.086 3.896 0.000

ANXPSYB 0.546 0.060 9.022 0.000

ANXSOMB 0.486 0.045 10.811 0.000

APPTB 0.399 0.059 6.741 0.000

GENERLB 0.519 0.043 12.137 0.000

SEXINTB 0.268 0.062 4.345 0.000

HYPCHDB 0.328 0.077 4.258 0.000

WGTLSB 0.352 0.089 3.964 0.000

FB2 BY

DEPMDB 0.342 0.071 4.784 0.000

GUILTB 0.130 0.070 1.847 0.065

SUCDB 0.260 0.078 3.329 0.001

EARLYB -0.018 0.222 -0.081 0.936

ISMMIDB 0.072 0.251 0.286 0.775

LATEB 0.043 0.188 0.231 0.817

WKACTB 0.322 0.101 3.181 0.001

RTARDB 0.601 0.079 7.621 0.000

AGITB -0.366 0.077 -4.743 0.000

ANXPSYB -0.003 0.027 -0.100 0.920

ANXSOMB 0.008 0.080 0.102 0.918

APPTB -0.070 0.164 -0.430 0.667

GENERLB 0.069 0.110 0.627 0.531

SEXINTB 0.122 0.085 1.443 0.149

HYPCHDB -0.106 0.089 -1.186 0.236

WGTLSB -0.351 0.197 -1.786 0.074

*4 Factor model

MODEL RESULTS

Two-Tailed

Estimate S.E. Est./S.E. P-Value

FB1 BY

DEPMDB 0.626 0.058 10.871 0.000

GUILTB 0.468 0.060 7.765 0.000

SUCDB 0.637 0.062 10.260 0.000

EARLYB -0.002 0.061 -0.029 0.977

ISMMIDB 0.083 0.075 1.115 0.265

LATEB 0.024 0.074 0.320 0.749

WKACTB 0.268 0.053 5.023 0.000

RTARDB -0.018 0.017 -1.020 0.308

AGITB 0.130 0.092 1.408 0.159

ANXPSYB 0.192 0.084 2.299 0.022

ANXSOMB -0.030 0.063 -0.470 0.638

APPTB -0.002 0.049 -0.038 0.970

GENERLB 0.156 0.086 1.806 0.071

SEXINTB 0.339 0.074 4.582 0.000

HYPCHDB -0.028 0.068 -0.420 0.675

WGTLSB -0.103 0.109 -0.945 0.345

FB2 BY

DEPMDB -0.047 0.044 -1.083 0.279

GUILTB -0.001 0.056 -0.011 0.991

SUCDB 0.008 0.039 0.197 0.844

EARLYB 0.527 0.060 8.791 0.000

ISMMIDB 0.660 0.067 9.801 0.000

LATEB 0.499 0.069 7.274 0.000

WKACTB 0.160 0.080 2.001 0.045

RTARDB 0.017 0.016 1.030 0.303

AGITB 0.133 0.148 0.897 0.370

ANXPSYB -0.111 0.193 -0.573 0.566

ANXSOMB 0.079 0.183 0.430 0.667

APPTB 0.428 0.133 3.212 0.001

GENERLB 0.217 0.164 1.325 0.185

SEXINTB 0.026 0.073 0.357 0.721

HYPCHDB -0.091 0.169 -0.541 0.589

WGTLSB 0.593 0.166 3.578 0.000

FB3 BY

DEPMDB 0.055 0.076 0.727 0.467

GUILTB 0.033 0.060 0.550 0.582

SUCDB -0.120 0.081 -1.485 0.138

EARLYB -0.067 0.138 -0.485 0.628

ISMMIDB -0.122 0.182 -0.674 0.500

LATEB -0.034 0.124 -0.273 0.785

WKACTB 0.277 0.143 1.946 0.052

RTARDB 0.409 0.192 2.130 0.033

AGITB 0.041 0.057 0.724 0.469

ANXPSYB 0.583 0.069 8.448 0.000

ANXSOMB 0.666 0.093 7.197 0.000

APPTB 0.263 0.150 1.755 0.079

GENERLB 0.437 0.139 3.142 0.002

SEXINTB 0.004 0.079 0.048 0.962

HYPCHDB 0.487 0.122 3.973 0.000

WGTLSB 0.084 0.103 0.813 0.416

FB4 BY

DEPMDB -0.071 0.055 -1.285 0.199

GUILTB 0.090 0.068 1.314 0.189

SUCDB 0.024 0.034 0.694 0.487

EARLYB 0.017 0.058 0.285 0.776

ISMMIDB -0.058 0.059 -0.973 0.331

LATEB -0.049 0.069 -0.709 0.478

WKACTB -0.253 0.058 -4.374 0.000

RTARDB -0.749 0.095 -7.924 0.000

AGITB 0.490 0.082 5.938 0.000

ANXPSYB 0.099 0.079 1.247 0.213

ANXSOMB -0.041 0.059 -0.692 0.489

APPTB 0.070 0.071 0.993 0.321

GENERLB -0.028 0.040 -0.718 0.473

SEXINTB 0.033 0.076 0.432 0.666

HYPCHDB 0.111 0.098 1.133 0.257

WGTLSB 0.384 0.100 3.862 0.000

*bi-3factor model

MODEL RESULTS

Two-Tailed

Estimate S.E. Est./S.E. P-Value

HAMB BY

DEPMDB 0.611 0.058 10.510 0.000

GUILTB 0.446 0.065 6.879 0.000

SUCDB 0.609 0.079 7.677 0.000

EARLYB -0.074 0.221 -0.334 0.739

ISMMIDB -0.007 0.303 -0.022 0.983

LATEB -0.042 0.224 -0.187 0.851

WKACTB 0.250 0.103 2.419 0.016

RTARDB 0.019 0.182 0.105 0.917

AGITB 0.083 0.136 0.611 0.542

ANXPSYB 0.198 0.180 1.097 0.273

ANXSOMB -0.034 0.119 -0.284 0.776

APPTB -0.061 0.119 -0.514 0.607

GENERLB 0.124 0.074 1.681 0.093

SEXINTB 0.320 0.078 4.099 0.000

HYPCHDB -0.018 0.194 -0.093 0.926

WGTLSB -0.196 0.181 -1.084 0.278

FB1 BY

DEPMDB 0.173 0.113 1.526 0.127

GUILTB 0.151 0.074 2.059 0.039

SUCDB 0.099 0.178 0.557 0.577

EARLYB 0.398 0.214 1.863 0.062

ISMMIDB 0.496 0.322 1.542 0.123

LATEB 0.405 0.220 1.840 0.066

WKACTB 0.405 0.085 4.768 0.000

RTARDB 0.301 0.158 1.906 0.057

AGITB 0.172 0.172 1.002 0.316

ANXPSYB 0.364 0.328 1.110 0.267

ANXSOMB 0.522 0.291 1.796 0.073

APPTB 0.543 0.056 9.742 0.000

GENERLB 0.532 0.119 4.474 0.000

SEXINTB 0.118 0.095 1.245 0.213

HYPCHDB 0.252 0.324 0.778 0.436

WGTLSB 0.527 0.098 5.396 0.000

FB2 BY

DEPMDB 0.088 0.089 0.985 0.325

GUILTB -0.066 0.070 -0.945 0.345

SUCDB -0.015 0.086 -0.174 0.862

EARLYB -0.046 0.085 -0.547 0.585

ISMMIDB 0.012 0.060 0.207 0.836

LATEB 0.018 0.074 0.247 0.805

WKACTB 0.252 0.068 3.723 0.000

RTARDB 0.713 0.101 7.039 0.000

AGITB -0.444 0.074 -6.013 0.000

ANXPSYB -0.031 0.039 -0.809 0.419

ANXSOMB 0.088 0.060 1.457 0.145

APPTB -0.063 0.084 -0.753 0.452

GENERLB 0.056 0.056 0.994 0.320

SEXINTB -0.022 0.075 -0.292 0.771

HYPCHDB -0.057 0.083 -0.691 0.489

WGTLSB -0.374 0.113 -3.307 0.001

FB3 BY

DEPMDB 0.051 0.167 0.306 0.759

GUILTB 0.073 0.119 0.616 0.538

SUCDB -0.063 0.198 -0.320 0.749

EARLYB -0.313 0.312 -1.004 0.315

ISMMIDB -0.450 0.342 -1.314 0.189

LATEB -0.301 0.276 -1.092 0.275

WKACTB 0.018 0.130 0.137 0.891

RTARDB -0.028 0.115 -0.240 0.810

AGITB 0.168 0.156 1.077 0.282

ANXPSYB 0.519 0.197 2.641 0.008

ANXSOMB 0.417 0.355 1.173 0.241

APPTB -0.004 0.353 -0.012 0.990

GENERLB 0.192 0.295 0.651 0.515

SEXINTB 0.012 0.097 0.123 0.902

HYPCHDB 0.440 0.197 2.230 0.026

WGTLSB -0.089 0.430 -0.208 0.835

*5 Factor model

MODEL RESULTS

Two-Tailed

Estimate S.E. Est./S.E. P-Value

FB1 BY

DEPMDB 0.656 0.056 11.631 0.000

GUILTB 0.462 0.058 7.937 0.000

SUCDB 0.638 0.060 10.566 0.000

EARLYB -0.032 0.053 -0.596 0.551

ISMMIDB 0.051 0.053 0.958 0.338

LATEB 0.015 0.058 0.257 0.797

WKACTB 0.276 0.058 4.802 0.000

RTARDB -0.007 0.025 -0.287 0.774

AGITB 0.119 0.084 1.426 0.154

ANXPSYB 0.201 0.068 2.959 0.003

ANXSOMB -0.032 0.063 -0.504 0.614

APPTB 0.003 0.059 0.053 0.957

GENERLB 0.175 0.077 2.263 0.024

SEXINTB 0.333 0.077 4.331 0.000

HYPCHDB -0.034 0.044 -0.789 0.430

WGTLSB -0.017 0.019 -0.865 0.387

FB2 BY

DEPMDB -0.126 0.063 -1.993 0.046

GUILTB 0.034 0.055 0.613 0.540

SUCDB 0.062 0.065 0.954 0.340

EARLYB 0.563 0.071 7.958 0.000

ISMMIDB 0.682 0.064 10.655 0.000

LATEB 0.429 0.073 5.855 0.000

WKACTB 0.019 0.039 0.493 0.622

RTARDB 0.060 0.062 0.980 0.327

AGITB 0.002 0.074 0.028 0.978

ANXPSYB -0.222 0.085 -2.600 0.009

ANXSOMB -0.090 0.093 -0.961 0.336

APPTB 0.284 0.084 3.365 0.001

GENERLB 0.032 0.062 0.521 0.602

SEXINTB -0.023 0.075 -0.314 0.754

HYPCHDB 0.033 0.041 0.791 0.429

WGTLSB 0.032 0.030 1.054 0.292

FB3 BY

DEPMDB 0.023 0.048 0.472 0.637

GUILTB 0.033 0.066 0.496 0.620

SUCDB -0.121 0.083 -1.448 0.148

EARLYB 0.020 0.069 0.284 0.777

ISMMIDB -0.017 0.051 -0.328 0.743

LATEB 0.026 0.076 0.341 0.733

WKACTB 0.292 0.146 1.993 0.046

RTARDB 0.402 0.251 1.599 0.110

AGITB 0.049 0.115 0.427 0.669

ANXPSYB 0.551 0.060 9.106 0.000

ANXSOMB 0.688 0.086 8.046 0.000

APPTB 0.304 0.077 3.969 0.000

GENERLB 0.433 0.110 3.931 0.000

SEXINTB 0.007 0.080 0.082 0.935

HYPCHDB 0.533 0.178 3.000 0.003

WGTLSB 0.021 0.015 1.437 0.151

FB4 BY

DEPMDB -0.042 0.044 -0.949 0.343

GUILTB 0.059 0.060 0.984 0.325

SUCDB 0.011 0.038 0.286 0.775

EARLYB 0.031 0.058 0.530 0.596

ISMMIDB -0.060 0.051 -1.178 0.239

LATEB -0.007 0.066 -0.113 0.910

WKACTB -0.221 0.090 -2.469 0.014

RTARDB -0.699 0.100 -7.012 0.000

AGITB 0.473 0.075 6.307 0.000

ANXPSYB 0.049 0.054 0.901 0.368

ANXSOMB -0.063 0.086 -0.731 0.465

APPTB 0.108 0.094 1.144 0.253

GENERLB -0.009 0.069 -0.134 0.894

SEXINTB 0.014 0.077 0.185 0.853

HYPCHDB 0.041 0.038 1.079 0.281

WGTLSB 0.722 0.225 3.212 0.001

FB5 BY

DEPMDB 0.099 0.084 1.171 0.241

GUILTB -0.112 0.077 -1.452 0.146

SUCDB -0.092 0.093 -0.984 0.325

EARLYB -0.049 0.075 -0.650 0.516

ISMMIDB 0.007 0.036 0.199 0.843

LATEB 0.137 0.087 1.580 0.114

WKACTB 0.190 0.077 2.474 0.013

RTARDB 0.036 0.030 1.198 0.231

AGITB 0.059 0.081 0.733 0.463

ANXPSYB -0.059 0.061 -0.972 0.331

ANXSOMB 0.022 0.071 0.305 0.761

APPTB 0.107 0.105 1.014 0.311

GENERLB 0.153 0.094 1.636 0.102

SEXINTB 0.061 0.064 0.955 0.340

HYPCHDB -0.511 0.148 -3.447 0.001

WGTLSB 0.878 0.257 3.421 0.001
