# Supplementary material for: The bi-factor structure of the 17-item Hamilton Depression Rating Scale in persistent major depression; dimensional measurement of outcome
Source: PLoS One. 2020 Oct 26;15(10):e0241370. doi: 10.1371/journal.pone.0241370 (PMC7588071; doi:10.1371/journal.pone.0241370)
Supplement: Attachment — Submitted filename: HDRS17.SL.docx [file pone.0241370.s003.docx]

Title: The Bi-Factor Structure of the 17-Item Hamilton Depression Rating Scale with Persistent Major Depression Disorder in Specialist Mental Health Services.

**Summary:** The authors of this study explored the 17-item Hamilton Depression Rating Scale (HDRS-17) by examining the dimensionality of this measure with individuals diagnosed with moderately severe persistent major depressive disorder (PMDD). Results reported a bi-factor structure of two group factors (e.g., insomnia worry and retardation-agitation) that were stable over a 12-month period. The authors suggested that the HDRS-17 is a valid measure of depression for patients with moderately severe depression.

Below I summarize my comments and concerns:

1) The authors identified individuals recruited in specialist mental health setting. It would be beneficial in understanding this population. Prevalence and severity of depression would help to identify why other measures were not chooses such as the BDI-II or the PHQ-9.
2) The authors mention inconsistencies of the HDRS17 factor structures even when compared to psychiatric interviews. The study’s methodology consisted of the Structured Clinical Interview (SCID) for DSM-IV. Analytical techniques using the SCID and the HDRS could have showed some consistency and validity of the data in terms of construct validity and reliability of the measure. There feels to be some missing processes known to be influential on the area of reported research. The study would benefit from analyses of reliability and validity across time stamps.
3) The authors evidenced fairly steady drops in the level of severity of depression. It would be beneficial to understand if treatment setting was moderating or mediating these values. Treatment with combination of CBT and psychopharmacological agent have shown the greatest reduction in depression severity. The authors study showed greater reduction of retardation-agitation factors score changes greater than worry-insomnia-factor. An elaboration of these results would be beneficial especially since factor loading of retardation-agitation are significant for depressed mood, suicidal thoughts, and agitations. It would have also been helpful if the results were comparable to other depression measures such as the PHQ-9 and BDI-II, and gain a better understanding of measurement precision and score comparability. The study in limited to the HDRS17 and it is difficult to really assess and control that the HDRS is capturing the whole spectrum of depression and it would be beneficial to apply some classical item response analysis.
4) Based on the above response and the novelty of the research study and inconsistencies related to the HDRS. The title of this study would be best fitted as a preliminary study of results.
