# Supplementary material for: The bi-factor structure of the 17-item Hamilton Depression Rating Scale in persistent major depression; dimensional measurement of outcome
Source: PLoS One. 2020 Oct 26;15(10):e0241370. doi: 10.1371/journal.pone.0241370 (PMC7588071; doi:10.1371/journal.pone.0241370)
Supplement: Attachment — Submitted filename: Response to reviewers bi-factor HDRS-17.docx [file pone.0241370.s004.docx]

Response to PLOS ONE reviewers.

Reviewer comments on normal font and authors’ replies in bold and italics.

Reviewer #1: I welcome the initiative of the authors to explore psychometric properties of the HDRS17 as, despite generally recognized shortcomings of this scale, it is the gold standard for measuring depression. The manuscript is well written and I agree with all of the strengths outlined on lines 295-304. As a manuscript about the dimensionality of the HDRS17 I have little to criticize. However, it is a grave scientific weakness of the paper that it makes wide claims about valid measurement, which implies much more than dimensionality. As this issue is so pivotal in this paper, and as it is bound to mislead the readership of the journal on a very central issue to psychometrics, I will focus on this issue in the following.

Through the paper, one finds an apparent conflation between the summary scale score — i.e. they official, conventional and very simple way of deriving a single total score for the whole HDRS17 — and the highly elaborate, multidimensional person estimates derived from the structural model. We see a hint of this in the introduction, where the authors express concern that the total score of the HDRS17 may not measure the severity of a general depression factor. Yet they show no interest in testing a unidimensional measure. In the discussion (line 281), the authors state that "Such consistency in the factor structure of the HDRS17 [across two samples] would suggest that the HDRS17 is a valid measure of the general severity of depression in such settings". Here the authors again disregard the fact that the HDRS17 is conventionally scored based on the assumption of unidimensionality. However, the most manifest collision between the two confused concepts — the HDRS as a summary scale and the HDRS as it is modelled in ESEM — is found in the conclusions, line 319: "The total score of items on this measure [the HDRS17] are valid at each time point and over time". This is fundamentally wrong; a total score cannot be valid if it reflects more than one dimension. Also, the authors use a model without tau-equivalence, so their claim does not apply to the raw total score of the HDRS17, placing their validity claims far afield from the actual use of the HDRS17. Additionally, a valid total score builds on the assumptions of local independence and the absence of differential item functioning.

If the authors wish to say anything about the validity of the raw total score of the HDRS-17, they need to test (or at least evaluate) all the above assumptions. If, on the other hand, the authors wish to revolutionize the way the HDRS-17 is used, they need to explain to the reader how their model can be used for the purpose of valid measurement (i.e. entering every item score into a computer program which will then return three measures for each individual) and discuss benefits and limitations of this use over alternative uses (e.g. the conventional HDRS-17 summary score as well as shortened subscales with the potential for a statistically sufficient raw score, such as the HamiltonD6/melancholia subscale). Alternatively, if the authors are only concerned with the dimensionality of the scale, they should clearly state this as their aim and they should clarify to the reader that their results do not support most of the assumptions underlying the current use of the HDRS17.

***We are grateful for the comments of reviewer 1, which stimulated considerable further thought. Following this we have substantially rewritten the introduction (paragraphs 2-5 and 7-9 of introduction) and discussion ( paragraphs 1-6, 6 and conclusion) sections, focusing on the factor structure of the scale, as a multidimensional measure. In keeping with this we have removed the previous references to ‘validity’ of the total score and have also removed the other specific lines identified.***

***We have presented our results asa dimensional approach to the measurment of outcome in one UK cohort of patients with moderate-severe persistent major depression and made reference in the discussion to the importance of replication within other similar cohorts; and that results cannot be assumed to generalise to other less severe or persistent populations in other settings (such as primary care), referencing further work on this.***

***Given the need for replication of the factor structure of the HDRS-17, we thought it important to also explicitly state that findings reported on change over 12 months are exploratory and have updated Table 5 to give further detail. We discuss some of the problems involved in operationalising this multidimensional model and the importance of replicating the bi-factor structure as a necessary prelude to this. With all of this in mind, as Reviewer 1 intimates, we are not in a position to revolutionize current use of the HDRS-17. We have instead made more modest calls for further work towards replication of our findings in similarly defined clinical groups (more severe persistent major depression). We have also discussed reasons why we cannot assume the observed factor structure to hold in other less severe, less persistent and likely less complex groups.***

***Holding to the limitations above, we have also given some greater salience to comparisons with current 6-item scales (of Bech and Maier), including the different ways these scales were derived and some of the similarities in item content with the current model. Given the importance of replicating our current findings we have not though gone any further to suggest our bi-factor model is used in preference to these scales.***

***As a result of these comments, the paper has been substantially re-written, particularly through the introduction and discussion sections and the line numbers have therefore changed but all quotes referenced by Reviewer 1 have been removed or substantially amended. Table 5 in the results section has also been re-drawn in keeping with the early stage of findings here and the more exploratory approach to what change over 12 months represents within this model. We think the manuscript is improved by these changes and are grateful to reviewer 1.***

Reviewer 2

**Summary:** The authors of this study explored the 17-item Hamilton Depression Rating Scale (HDRS-17) by examining the dimensionality of this measure with individuals diagnosed with moderately severe persistent major depressive disorder (PMDD). Results reported a bi-factor structure of two group factors (e.g., insomnia worry and retardation-agitation) that were stable over a 12-month period. The authors suggested that the HDRS-17 is a valid measure of depression for patients with moderately severe depression.

Below I summarize my comments and concerns:

1) The authors identified individuals recruited in specialist mental health setting. It would be beneficial in understanding this population. Prevalence and severity of depression would help to identify why other measures were not chooses such as the BDI-II or the PHQ-9.

***We are grateful to Reviewer 2 and will answer this in parts:***

1. ***All subjects were recruited from community mental health settings in the UK and with only half of these receiving additional collaborative care and the others continuing to receive general community mental health care. We have clarified this in the text ( method lines 121-123).***
2. ***Inclusion criteria from the initial study stated depression of at least moderate clinical severity, operationalised as HDRS-17 of 16 or over. We chose a mixture of clinician-rated outcome measures (CROMs; HDRS-17) and patient-rated outcome measures (PROMs; QIDS, PHQ-9 and BDI-1) considering that whilst we might expect a moderate-strong correlation, they could not necessarily be relied on to measure the same construct. The study team was aware that the different types of measure (CROMs and PROMs) might hold different perspectives and different points of reference (e.g. a clinical range vs personal experience) and so we considered them complimentary rather than replicative measures, both relevant to the study of Persistent Major Depression. The degree of correlation between CROMs and PROMs in this population is an interesting question and we have answered in more detail below, under point 3, comparing our findings with recently published material, though we have chosen not to include this data in the current manuscript in order to maintain a clearer focus on dimensionality of the HDRS17. We have though clarified some further detail from the initial study in the revised text ( methods lines 125-136).***
2) The authors mention inconsistencies of the HDRS17 factor structures even when compared to psychiatric interviews. The study’s methodology consisted of the Structured Clinical Interview (SCID) for DSM-IV. Analytical techniques using the SCID and the HDRS could have showed some consistency and validity of the data in terms of construct validity and reliability of the measure. There feels to be some missing processes known to be influential on the area of reported research. The study would benefit from analyses of reliability and validity across time stamps.

***We have removed the reference to inconsistency with psychiatric interview, including any structured interview (e.g. SCID). We did not have strong enough data from the SCID to assess correlation but as shown in Table 1 (below), there was a moderate-strong correlation across the 3 time-points measured (Baseline, 6 and 12 months) between the HDRS-17 and three Patient Report Outcome Measures (PHQ-9, BDI, QIDS). The correlation was higher (within the moderate-strong range) for later timepoints, consistent with recent findings from Hershenberg et al. (2020) who showed greater correlation for patients under longer-term care (vs. patients at first contact). One might speculate on the reasons for greater agreement between clinician and patient outcome measures over time, such as developing trusting relationships, but the phenomenon seems consistent with other recent literature. Although we agree this is an important area, we would rather keep the current focus on factor structure and consider correlations in a later paper***.

.

3) The authors evidenced fairly steady drops in the level of severity of depression. It would be beneficial to understand if treatment setting was moderating or mediating these values. Treatment with combination of CBT and psychopharmacological agent have shown the greatest reduction in depression severity. The authors study showed greater reduction of retardation-agitation factors score changes greater than worry-insomnia-factor. An elaboration of these results would be beneficial especially since factor loading of retardation-agitation are significant for depressed mood, suicidal thoughts, and agitations. It would have also been helpful if the results were comparable to other depression measures such as the PHQ-9 and BDI-II, and gain a better understanding of measurement precision and score comparability. The study in limited to the HDRS17 and it is difficult to really assess and control that the HDRS is capturing the whole spectrum of depression and it would be beneficial to apply some classical item response analysis.

***We are grateful to Reviewer 2 for this comment and will answer in several parts to this, which are addressed separately below:***

1. ***The specialist treatment (specialist CBT and psychopharmacology) was not dissociable from the treatment setting, indeed the integration of these approaches through ‘collaborative care’ was integral to the main study design. This intervention (collaborative care delivery of specialist CBT and psychopharmacology) was associated with measurable changes during treatment (not present at baseline) and we therefore consider treatment as a mediator (rather than moderator) of outcome. Although the suggestion of a mediator analysis is interesting, it is outside the scope of the current study, on HDRS-17 dimensionality and we are reluctant to change the focus of the current paper. However, we thank reviewer 2 for stimulating thought on this and will consider a future path analysis, assessing correlated change of transdiagnostic measures with the separate HDRS factors shown here.***
2. ***We agree the greater reduction in the retardation-agitation factor is of relevance clinically and have added to both the discussion (lines 250-275) and conclusion sections to reflect this. We have given further detail on this change in factor score over 12 months (Table 5) but have discussed it as an exploratory analysis; with need to replicate the basic bi-factor structure and then work towards operationalizing findings.***
3. ***We have performed a correlation analysis, presented below in Table 1, which shows significant and moderate-strong correlation between the observer rated HDRS-17 and all patient completed outcome measures used in this study (PHQ-9, BDI-1 and QIDS, at each time point). Correlation shown in this analysis was similar to that reported in another recent publication in a Treatment Resistant Depression population (Hershenberger et al, 2020), particularly for the closest comparison (at baseline comparison, before any clinical improvement).***

Table 1

|  | HDRS-17 | Current Study |  | HDRS-17 |
| --- | --- | --- | --- | --- |
|  | Baseline | 6 months | 12 months | Reported* |
| BDI-1 | 0.56 | 0.75 | 0.78 | 0.65 |
| QIDS-SR | 0.55 | 0.69 | 0.76 | 0.57 |
| PHQ-9 | 0.49 | 0.68 | 0.79 | Not Available |

*From Hershenberg et al, Journal of Affective Disorders, April 2020

4. ***Our study aimed to assess the dimensionality of the HDRS-17 measure with preliminary evidence of a bi-factor structure fitting the data well. We did not hypothesise that the HDRS-17 measures a single concept but conducted an exploratory analysis that identified a multidimensional construct, with specific factor scores indicating the severity of relevant aspects of depression. In the initial RCT, we did not hypothesise that the HDRS-17 would capture the whole spectrum of depression and included a number of scales therefore (PHQ-9, BDI and QIDS-SR) as complimentary, rather than strictly replicative measures that might give a fuller understanding, both from different perspectives (patient vs clinician) and slightly different concepts of ‘depression’ (for example including appetite gain, as well as loss in QIDS-SR). Although we accept the point made here, again we would rather keep the focus of the current paper on the important issue of the dimensionality of the HDRS-17.***
4) Based on the above response and the novelty of the research study and inconsistencies related to the HDRS. The title of this study would be best fitted as a preliminary study of results.

***Thank you for this recommendation, which we have accepted in full.***

***The full title now reads “The bi-factor structure of the 17-item Hamilton Depression Rating Scale in moderate to severe persistent major depression; dimensional measurement of outcome”***

***We think this better represents the early findings reported here that need replication and we’re grateful to Reviewer 2 for the stimulating comments, which have hopefully improved the manuscript.***
