# Supplementary material for: The bi-factor structure of the 17-item Hamilton Depression Rating Scale in persistent major depression; dimensional measurement of outcome
Source: PLoS One. 2020 Oct 26;15(10):e0241370. doi: 10.1371/journal.pone.0241370 (PMC7588071; doi:10.1371/journal.pone.0241370)
Supplement: Attachment — Submitted filename: Response to reviewers bi-factor HDRS-17.docx [file pone.0241370.s005.docx]

Response to Reviewer 1

1. We were asked to deal with the potential validity of the bi-factor model in the context of the Melancholia (Bech, Maier) subscale - that this should not be postponed and the specific example of negative loadings needs to be addressed, including whether person estimates for these traits can be used for outcome measurement?

We re-considered the implications of the HDRS_17_ bi-factor structure shown in our analysis. We agree that this is not appropriate for use as an outcome measure in the moderate-severe PMDD group studied here, given that it is not a unidimensional measure and that domain factors include negatively loading items. In light of this and previous evidence on the HDRS_6_ (Bech), we conducted a further post-hoc analysis to assess the dimensionality of the HDRS_6_ subscale in this group. This analysis is given below and included as a post-hoc analysis in our updated results section. It shows a significant positive loading of all 6 items (0.396 – 0.544, p < 0.01) for this moderate-severe PMDD group, with time invariance. Considering this fuller evidence within the wider context advocated by Reviewer 1, we have revised our discussion, proposing the HDRS_6_ as an outcome measure in moderate-severe PMDD. The HDRS_17_ may retain a clearer role in psychiatric assessment where its more complex structure identifies types of PMDD, acting as a potential predictor of effective treatment strategies for settled states of depression (e.g. for agitated vs. retarded depression).

2. Address why we have related the melancholia subscale to the ‘retardation-agitation’ domain rather than the general factor, when 5 out of 6 items on the melancholia subscale (Bech) display highest loadings on the general factor?

The HDRS-6 comprises *depressed mood, anhedonia (work and interests), guilt, fatigue, psychological anxiety and psychomotor retardation* (Bech 1981); and as Reviewer 1 points out, 5 of these 6 items showed their highest loading on the general factor, rather than either of the domain factors. Prompted by this, we ran a further analysis investigating the structure of the HDRS_6_ in our PMDD cohort, specifically to investigate whether this showed a unidimensional structure and time-invariance. For this reason (and because it is not plausible to do exploratory analysis testing model with 1 to 3 factors on a 6-item scale), we focused on testing the unidimensional factor structure only, i.e. we used a one factor model with results given below:

| Item | HDRS_6_ |
| --- | --- |
| Depressed Mood | .544* |
| Work and Interests | .526* |
| General Somatic (Tiredness) | .474* |
| Psychic Anxiety | .417* |
| Gulit Feelings | .396* |
| Psychomotor retardation | .407* |

* all loading estimates are statistically significant at p<0.01

The HDRS_6_ also showed stability over time:

**Table Bech scale Fitting indices of ME/I across measurement time**

| Model | χ^2^(df),p= | RMSEA | CFI | NNFI | ΔCFI | Δχ^2^(df),p= |
| --- | --- | --- | --- | --- | --- | --- |
| Configural | 193.266(120),0.000 | .057 | .934 | .913 |  |  |
| Scalar a | 459.218(165),0.000 | .098 | .736 | .755 |  | 259.790(45), p=0.000 |
| Scalar b | 229.625(146),0.000 | .055 | .925 | .921 | -.009 | 44.883(26), p=0.012 |

Note: scalar b model freed 24 of 55 (43%)threshold parameters

The unidimensionality, positive item loading and stability of the HDRS_6_ indicate it may be used as an outcome measure in moderate-severe PMDD. Additionally it is more parsimonious than the HDRS_17_, therefore quicker to administer and much more likely to be of clinical use as an outcome measure. We’re grateful for Reviewer 1 pointing us in this direction and have changed our discussion to indicate the potential use of the HDRS_17_ within assessment, with its more complex structure helping to predict effective treatment interventions; with evidence-based item-sets such as the HDRS_6_ used for outcome measurement. This approach seems consistent with Hamilton’s (1960) own assessment of the multidimensionality of the HDRS_17_ and its use in measuring settled states, rather than treatment change.

3. Did we include residual correlation parameters for the three insomnia related items?

No, as correlating residuals is generally not allowed when running SEM models and would only have been justifiable for repeated items or MTMM items when Items were analysed as a different set of factors.

4. Given the instability of the HDRS factor structure, will the bi-factor model change this?

No, we agree this won’t save the HDRS_17_ as a measure of outcome change. The bi-factor structure may remain useful to clinicians and researchers assessing settled states of depression (rather than assessing change in state) and we have added to the discussion of this.

5. Only the General factor can be used as an outcome measurement, with the subscales (containing neg loadings) only as predictor factors (e.g. for different types of interventions being more helpful) – given this why even stick with the particular set of questions in the HDRS-17?

We have carefully considered this. Given our results on the factor structure of the HDRS-17 in this well-defined clinical population (moderate-severe PMDD), we consider a more evidence-based position would be to recommend use item sets such as the HDRS_6_ in outcome measurement, with the HDRS_17_ having a more restricted use, within assessment of settled states (where the more complex structure may be an advantage in developing treatment strategies). We are very grateful for the helpful comments of Reviewer 1 in reconceptualising these results.
