# Supplementary material for: The bi-factor structure of the 17-item Hamilton Depression Rating Scale in persistent major depression; dimensional measurement of outcome
Source: PLoS One. 2020 Oct 26;15(10):e0241370. doi: 10.1371/journal.pone.0241370 (PMC7588071; doi:10.1371/journal.pone.0241370)
Supplement: Attachment — Submitted filename: Response to reviewers bi-factor HDRS-17Oct2020.docx [file pone.0241370.s006.docx]

**Response to reviewers.**

We are grateful for the further comments, which are addressed specifically below. Additionally, we have included the data requested.

Review Comments to the Author
6.	“I commend the authors on a well executed revision to their manuscript. My only additional request, is that the authors add a brief clarification in their manuscript, that they did not find any substantial residual correlations in the bi-factor model, as this would violate a basic assumption of the model”

We thank Reviewer 1 for the further comments. It is not a recommended convention to correlate error terms when preforming CFA and SEM [1-3] and we therefore set the correlation between item residuals as 0, as per Mplus default setting for ESEM. For complete clarity we have added to lines 174/5 in the text: *All ESEM models were performed using software Mplus 8 and in keeping with standard practice correlation between item residuals was set as 0.*

“Also, optionally, I may recommend the authors to state that their results for the HDRS-6 do not establish if the total score features statistical sufficiency”

We discussed this option within the team and whilst tempting, we concluded that additional comment on a post-hoc analysis would take the discussion too far from the central findings. However, in writing this we agree that future work with a main focus on statistical sufficiency (or indeed alternative item inclusion) is warranted.

1.	McDonald R, Ho M-H. Principles and practice in reporting Structural Equation Analyses. Psychological Methods. 2002;7:64-82. doi: 10.1037/1082-989X.7.1.64.

2.	Schreiber JB, Nora A, Stage FK, Barlow EA, King J. Reporting Structural Equation Modeling and Confirmatory Factor Analysis results: A review. The Journal of Educational Research. 2006;99(6):323-38. doi: 10.3200/JOER.99.6.323-338.

3.	Hermida R. The problem of allowing correlated errors in Structural Equation Modeling: Concerns and considerations. Computational Methods in Social Sciences. 2015;3:1-17.
